# Low-Dose Docetaxel Is Effective in Reducing Atherogenic Lipids and Atherosclerosis

**DOI:** 10.3390/ijms26041484

**Published:** 2025-02-11

**Authors:** Hong Y. Choi, Isabelle Ruel, Shiwon Choi, Iulia Iatan, Senna Choi, Jyh-Yeuan Lee, Jacques Genest

**Affiliations:** 1 Research Institute of the McGill University Health Centre, Montréal, QC H4A 3J1, Canada; isabelle.ruel@affiliate.mcgill.ca (I.R.); shiwon.choi@umontreal.ca (S.C.); iulia.iatan@mail.mcgill.ca (I.I.); senna.choi@mail.mcgill.ca (S.C.); jacques.genest@mcgill.ca (J.G.); 2 Department of Biochemistry, Microbiology, and Immunology, Faculty of Medicine, University of Ottawa, Ottawa, ON K1H 8M5, Canada; jyh-yeuan.lee@uottawa.ca

**Keywords:** atherosclerosis, cholesterol, high-density lipoprotein, desmocollin 1, docetaxel

## Abstract

High-density lipoprotein (HDL) particles form during cellular cholesterol removal, positioning HDL biogenesis as a potential strategy to combat atherosclerosis. We identified desmocollin 1 (DSC1) as a negative regulator of HDL biogenesis and discovered that docetaxel (DTX) effectively inhibits DSC1 activity. This study assessed the efficacy of DTX in reducing atherosclerosis in *ApoE*^−/−^ mice. After two weeks on a high-fat diet, mice were divided into baseline, vehicle-treated, and DTX-treated groups. Baseline mice were sacrificed at the end of the two weeks, while the other groups received a vehicle or DTX (1 μg/μL) via subcutaneously implanted osmotic pumps delivering 0.15 μL/h for six weeks, with the high-fat diet continued. The controlled drug delivery system maintained stable DTX blood concentrations (2.7–4.3 nM) over six weeks without hematologic toxicity. DTX treatment significantly reduced circulating atherogenic lipids, including triglycerides, non-esterified fatty acids, low-density lipoprotein cholesterol, and total cholesterol, while increasing the HDL cholesterol/total cholesterol ratio. These improvements were associated with significant reductions in atherosclerotic lesions in the aortic sinus and arch. Notably, these effects occurred without altering circulating inflammatory cytokine levels. These results demonstrate that DTX effectively reduces dyslipidemia-induced atherosclerosis. Its HDL-biogenic and anti-atherosclerotic effects establish DTX as a promising candidate for developing HDL-directed therapies for atherosclerosis.

## 1. Introduction

The buildup of cholesterol within arterial walls is the primary driver of atherosclerotic plaque formation. However, no current treatments effectively promote the removal of cholesterol from these plaques [1]. Excess cellular cholesterol is removed during the formation and maturation of high-density lipoprotein (HDL) particles, a process known as HDL biogenesis [2]. This process is the initial, rate-limiting step in the reverse cholesterol transport (RCT) pathway, where cholesterol is removed from atherosclerotic plaques and subsequently delivered to the liver for final excretion into the feces [3,4]. Impaired HDL biogenesis has been observed in various genetic and pathological conditions linked to an increased risk of atherosclerotic cardiovascular diseases (ASCVDs), including dyslipidemia, chronic kidney disease, and diabetes [5]. Despite extensive efforts, including the recent Phase III AEGIS-II trial of a novel apolipoprotein A-I (ApoA-I) formulation [6], clinical success in reducing ASCVD by enhancing HDL biogenesis and function remains elusive.

The adenosine triphosphate-binding cassette transporter A1 (ABCA1) is vital for transferring cellular cholesterol and phospholipids to ApoA-I, thereby leading to the formation of HDL particles [7]. Over the past few decades, strategies to enhance HDL biogenesis have focused on upregulating the expression and function of both ABCA1 and ApoA-I. While these intensive studies have deepened our understanding of the regulatory mechanism of these proteins, the complex and multi-layered nature of their regulation has made it challenging to specifically upregulate them for HDL biogenesis and to translate these findings into effective HDL-directed atheroprotective therapies [7,8].

In our quest to identify novel druggable targets, we isolated and characterized plasma membrane microdomains associated with HDL biogenesis. This unbiased approach led to the identification of desmocollin 1 (DSC1) as a new negative regulator of HDL biogenesis [9]. DSC1 prevents ABCA1-mediated HDL formation by binding and sequestering ApoA-I, making it the first known cell-surface protein whose inhibition can enhance HDL biogenesis [9]. To pharmacologically target DSC1-ApoA-I interactions, we virtually screened over 10 million chemical compounds and identified docetaxel (DTX), a Food and Drug Administration (FDA)-approved cancer chemotherapy drug, as a potent inhibitor [10]. DTX significantly enhanced HDL biogenesis at non-cytotoxic, low-nanomolar concentrations in human cells, including macrophages and smooth muscle cells (SMCs), two major cell types involved in the initiation and progression of atherosclerosis [2,10]. Additionally, DTX exerts atheroprotective effects by inhibiting the platelet-derived growth factor receptor-β signaling pathway in vascular SMCs [11], which drives SMC proliferation and migration [12,13]. Abnormal SMC proliferation, migration, and extracellular matrix synthesis within the arterial wall contribute to intimal thickening, a key event in the pathogenesis of atherosclerosis [14,15]. Given that the accumulation of cholesterol-loaded foam cells and the proliferation of SMCs in the arterial intima are biochemical hallmarks of atherosclerosis, DTX’s ability to enhance HDL biogenesis and suppress SMC proliferation presents considerable atheroprotective potential.

This study aimed to determine whether non-cytotoxic doses of DTX could reduce atherosclerosis. To test this, we employed an osmotic drug delivery system to maintain low-nanomolar concentrations of DTX consistently in the circulation of mice. Our findings suggest that low-nanomolar DTX treatment may offer a new therapeutic strategy for reducing dyslipidemia-induced atherosclerosis.

## 2. Results

### 2.1. Establishment of a DTX Administration Protocol for Atherosclerosis Studies in Mice

**Figure 1:**
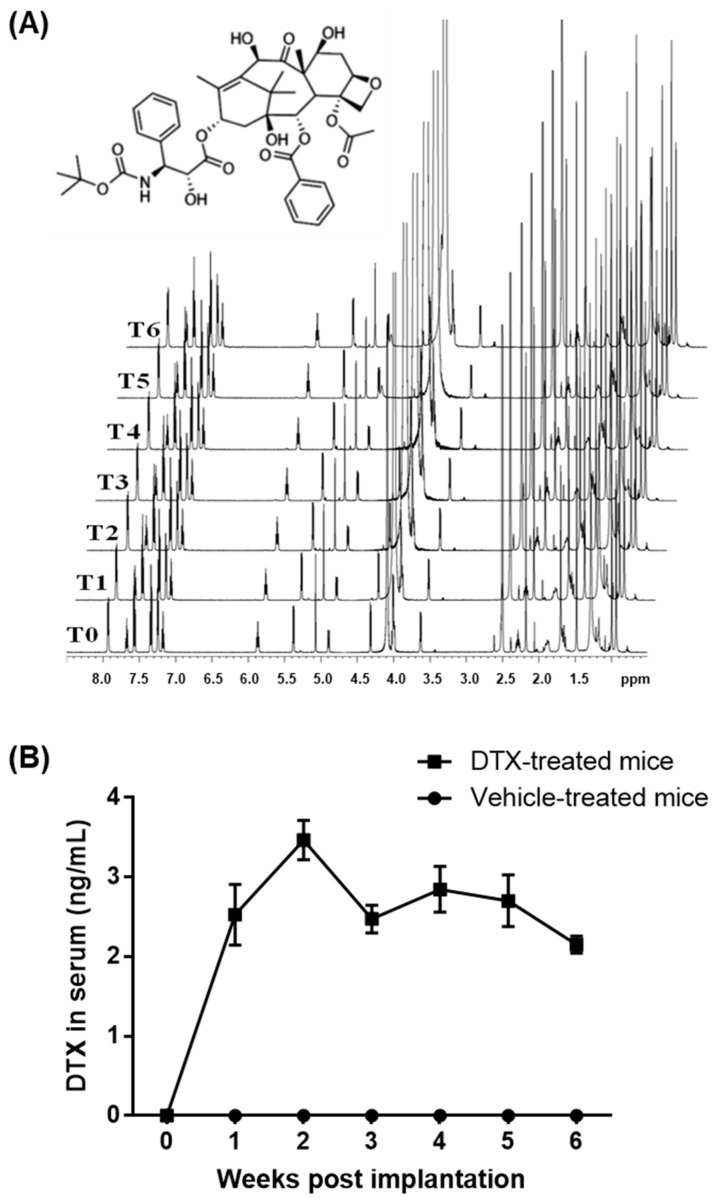
Stability and circulating concentrations of DTX. (**A**) Chemical structure of DTX and ^1^H NMR analysis of DTX stability: DTX was dissolved at 1 μg/μL in a 50% DMSO-D6 and 50% D_2_O solution and incubated at 37 °C for 6 weeks. Samples were collected at the start (time 0, T0) and weekly (T1–T6) for ^1^H NMR analysis. The spectra remained consistent throughout the incubation, indicating that DTX is chemically stable under these conditions. (**B**) DTX delivery and measurement of circulating DTX concentrations: The ALZET model 2006 pump, containing either DTX (1 μg/μL in a 50% DMSO and 50% PEG 300 solution) or the vehicle solution, was implanted subcutaneously into ten-week-old *ApoE*^−/−^ male mice (n = 5). Blood samples were collected prior to implantation and subsequently every week for 6 weeks to assess circulating DTX levels. Data values are presented as the mean ± SEM of measurements from the 5 mice.

DTX has shown HDL biogenic activity in human skin fibroblasts, human aortic SMCs, and differentiated human THP-1 macrophages at concentrations of 1–5 nM, without inducing cytotoxicity [2,10]. In contrast, the maximum chemotherapy doses of DTX in blood range from 1.9 to 5.1 μM [16,17]. These data suggest that non-cytotoxic, low-nanomolar concentrations of DTX may be used to prevent or treat atherosclerosis. To test this concept in *ApoE*^−/−^ mice, one of the most widely used animal models for dyslipidemia-induced atherosclerosis research [18,19], we employed an implantable osmotic pump (ALZET model 2006). Subcutaneous implantation of the pump enables continuous drug delivery at a constant rate of 0.15 μL/h for 6 weeks. This controlled release system provides an optimal method for administering precise, non-cytotoxic doses of DTX. To maintain continuous delivery of DTX in its chemically intact form, DTX must remain both soluble and chemically stable in the pump’s reservoir at body temperature for the entire 6-week duration. DTX is poorly soluble in water but dissolves well in dimethyl sulfoxide (DMSO). The pump is compatible with DMSO at concentrations of up to 50% in water or polyethylene glycol (PEG) (https://www.alzet.com/alzaid-chemical-compatibility-test-kit/) (accessed on 18 September 2022). However, PEG generates a broad range of proton signals that could interfere with DTX signals in proton nuclear magnetic resonance (^1^H NMR) spectroscopy, which analyzes proton behavior in a magnetic field to assess structural and compositional changes in organic compounds. To avoid this interference, we opted to use water for testing the chemical stability of DTX using ^1^H NMR spectroscopy. The maximum solubility of DTX in a 50% DMSO and 50% water mixture is approximately 1.3 μg/μL. For ^1^H NMR studies, we prepared a solution of 1 μg/μL DTX in a 50% deuterated DMSO (DMSO-D6) and 50% deuterated water (D_2_O) mixture. The solution was incubated at 37 °C for 6 weeks, with aliquots taken weekly for ^1^H NMR analysis to monitor any structural and compositional changes in DTX throughout the incubation period. The resulting ^1^H NMR spectra showed no notable variations, indicating that DTX remains chemically stable in the mixture at 37 °C for at least 6 weeks (Figure 1A).

PEG is commonly utilized in drug formulation to enhance the solubility of poorly water-soluble drugs, improve drug stability, and facilitate sustained or controlled drug release [20]. Therefore, we used PEG 300 instead of water to optimize DTX delivery with the ALZET model 2006 pump. The pump was filled with a solution containing 1 μg/μL of DTX in a 50% DMSO and 50% PEG 300 mixture. It was then equilibrated in a 0.9% saline solution at 37 °C for 60 h prior to being implanted subcutaneously between the scapulae of ten-week-old *ApoE*^−/−^ male mice. Blood samples were collected before implantation and weekly thereafter for six weeks to measure circulating DTX concentrations. Throughout the six-week period, the average DTX concentrations in the blood were maintained within the range of 2.15–3.47 ng/mL, corresponding to 2.7–4.3 nM (Figure 1B). These levels are comparable to HDL biogenic concentrations in cells (1–5 nM) and are approximately 1000 times lower than typical chemotherapy doses (1.9–5.1 μM) of DTX. This DTX administration protocol was therefore used to investigate the effects of DTX on atherosclerosis in *ApoE*^−/−^ mice.

### 2.2. DTX Exhibits No Hematologic Toxicity at Low-Nanomolar Concentrations

The main dose-limiting adverse effect of DTX treatment is hematologic toxicity [21,22]. To assess whether blood concentrations of 2.7–4.3 nM DTX in the *ApoE*^−/−^ mice caused hematologic toxicity, we collected whole blood samples at the end of the 6-week DTX administration period and conducted a complete blood count test. This test measured levels of hemoglobin, hematocrit, mean corpuscular volume, and mean corpuscular hemoglobin concentration, in addition to counting white blood cells, red blood cells, and platelets. All measurements were within normal ranges (Table 1) [23], and there were no statistically significant differences among the non-treated, vehicle-treated, and DTX-treated groups, indicating that the DTX concentrations did not cause hematologic toxicity.

### 2.3. DTX Improves the Circulating Lipid Profile

**Figure 2:**
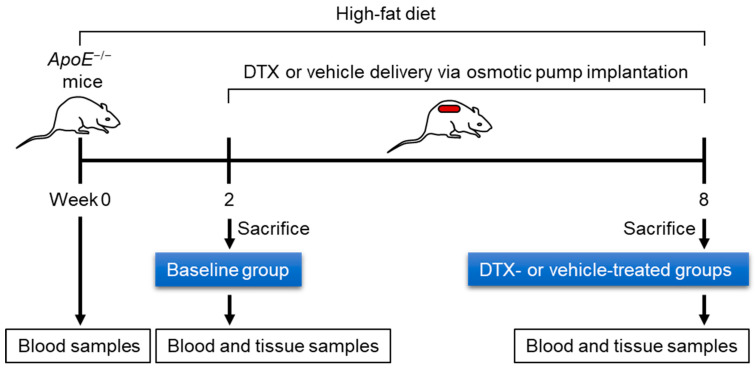
Experimental timeline for evaluating the effect of DTX on dyslipidemia-induced atherosclerosis. Ten-week-old *ApoE*^−/−^ male mice were fed an HF diet (21% fat and 0.2% cholesterol) for 8 weeks to induce dyslipidemia and atherosclerosis. During the final 6 weeks, the mice were treated with either DTX or a vehicle control. Blood samples were collected after a 4 h fast to measure fasting lipid levels. Upon sacrifice, the heart and aorta were harvested for the assessment of atherosclerosis development.

**Figure 3:**
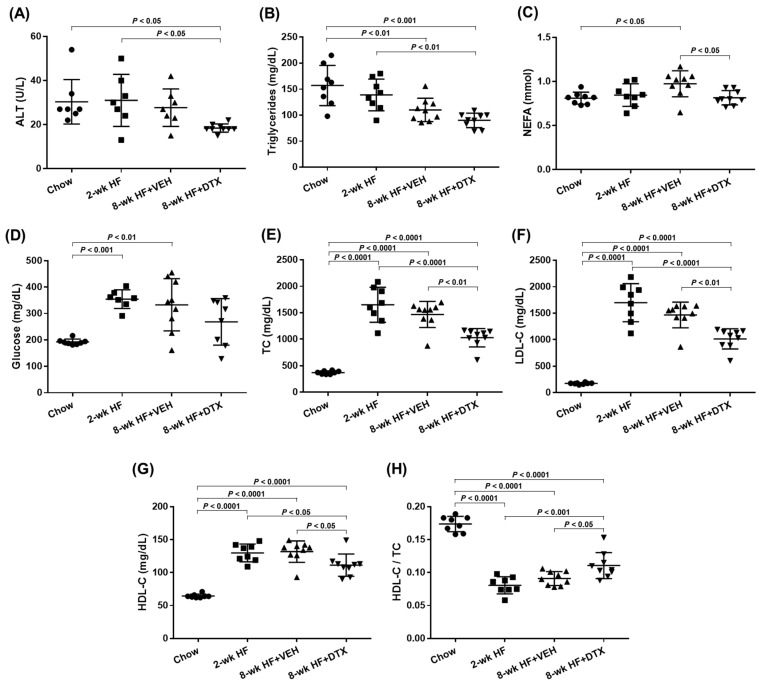
Serum levels of ALT, glucose, and lipids in *ApoE*^−/−^ mice. Ten-week (wk)-old *ApoE*^−/−^ male mice were fed an HF diet and treated according to the timeline outlined in Figure 2. Blood samples were collected from chow-fed mice (immediately before starting the HF diet), baseline group mice (2-wk HF), vehicle (VEH)-treated mice (8-wk HF + VEH), and DTX-treated mice (8-wk HF + DTX). Serum samples, separated from the blood, were analyzed to measure circulating levels of ALT (**A**), triglycerides (**B**), NEFA (**C**), glucose (**D**), TC (**E**), LDL-C (**F**), and HDL-C (**G**) and the HDL-C/TC ratio (**H**). Data are presented as scatter plots (n = 7–9) with mean ± standard deviation. Statistical analysis was performed using one-way ANOVA with Tukey’s post hoc test to adjust for multiple comparisons, with *p* < 0.05 considered statistically significant.

To investigate the effect of DTX on dyslipidemia-induced atherosclerosis, ten-week-old *ApoE*^−/−^ mice were first bled to measure circulating lipid levels while on a standard chow diet. The mice were then switched to a high-fat (HF) diet containing 21% fat and 0.2% cholesterol to induce dyslipidemia. After 2 weeks on the HF diet, one third of the mice were sacrificed to establish a baseline group. The remaining mice were divided into two groups and received subcutaneous implantation of an ALZET model 2006 pump loaded with either 1 μg/μL of DTX or a vehicle solution (50% DMSO and 50% PEG 300). These implanted mice continued on the HF diet for an additional 6 weeks before being sacrificed. Blood and tissue samples were collected at the end of the 2-week period (baseline group) and at 8 weeks (DTX- and vehicle-treated groups). Figure 2 illustrates the schematic timeline of this study. Serum samples separated from blood were analyzed for circulating lipid levels and other metabolic markers related to dyslipidemia. Serum alanine aminotransferase (ALT) levels are a biochemical marker used not only to detect drug-induced hepatotoxicity but also to predict hepatic steatosis [24,25]. The decreased ALT levels observed in the DTX-treated group suggest that DTX is not harmful to the liver and may have a positive effect on hepatic lipid metabolism (Figure 3A). DTX-treated mice showed reduced circulating triglyceride (TG) levels compared to the baseline group (Figure 3B), suggesting that DTX may lead to a reduction in hepatic TG synthesis and/or very-low-density lipoprotein secretion. DTX treatment normalized the elevated circulating non-esterified fatty acid (NEFA) levels observed in the vehicle-treated group (Figure 3C). Since high serum NEFA levels are associated with impaired glucose disposal and insulin resistance [26], DTX treatment correspondingly decreased serum glucose levels (Figure 3D). Circulating cholesterol levels, including total cholesterol (TC) (Figure 3E), LDL cholesterol (LDL-C) (Figure 3F), and HDL cholesterol (HDL-C) (Figure 3G), were all reduced in DTX-treated mice. As LDL particles are the primary carrier of cholesterol in the bloodstream and LDL-C is the most atherogenic lipid, these results strongly suggest that DTX may help reduce atherosclerosis. Although DTX treatment lowered all circulating cholesterol levels, it also resulted in an increased HDL-C/TC ratio (Figure 3H). This increase may be attributed to defective HDL catabolism or enhanced HDL biogenesis. As TC decreased in the DTX-treated group compared to the baseline group (Figure 3E), HDL-C also decreased (Figure 3G), suggesting that HDL catabolism likely occurred normally. Therefore, the increased HDL-C/TC ratio is most likely due to enhanced HDL biogenesis driven by DTX [10,27].

These results demonstrate that DTX improves the circulating lipid profile by reducing atherogenic lipids (TG, NEFA, TC, and LDL-C) and increasing the HDL-C/TC ratio. This suggests that DTX may be a potential treatment for atherogenic dyslipidemia.

### 2.4. DTX Does Not Directly Affect the Modulation of Inflammatory Cytokines

Atherosclerosis is recognized as a chronic inflammatory disease driven by the abnormal accumulation of atherogenic lipids within the arterial walls. Throughout the disease’s progression, these lipids and inflammatory cytokines interact, thus influencing each other in multiple ways [28,29]. To examine whether DTX treatment affects circulating inflammatory cytokine levels, plasma samples were separated from blood and used to measure 25 cytokines, including both pro-inflammatory cytokines (IL-1β, IL-6, IL-12, IL-18, IL-22, IL-23, IFNγ, and TNFα) and anti-inflammatory cytokines (IL-4, IL-10, IL-19, and IL-33) [30,31]. Among the twenty-five cytokines, seven were below the detection limit: IL-1β (<8.50 pg/mL), IL-2 (<2.41 pg/mL), IL-4 (<0.93 pg/mL), IL-12p70 (<11.58 pg/mL), IL-33 (<41.08 pg/mL), GM-CSF (<19.36 pg/mL), and IFNγ (<3.97 pg/mL). Of the remaining 18 cytokines measured, 17—IL-5, IL-6, IL-10, IL-13, IL-15, IL-17A, IL-17F, IL-21, IL-22, IL-23, IL-25/IL-17E, IL-27, IL-28B, IL-31, TNFα, CD40L, and MIP-3α/CCL20—did not show statistically significant differences among the chow-fed, baseline, vehicle-treated, and DTX-treated groups (Supplemental Figure S1, panels A to Q). The levels of TNFβ were significantly higher in the baseline, vehicle-treated, and the DTX-treated groups compared to the chow-fed group, with no significant differences observed between the baseline, vehicle-treated, and the DTX-treated groups (Supplemental Figure S1, panel R). Although TNFβ shares structural similarities with the major pro-inflammatory and atherogenic cytokine TNFα, their biological effects differ, and the role of TNFβ in atherosclerosis remains controversial.

*ApoE*^−/−^ mice fed an HF diet for 8 weeks develop early-stage atherosclerosis [32]. The cytokine levels measured suggest that inflammation may not be a significant factor in the early stages of dyslipidemia-induced atherosclerosis, and DTX does not appear to directly influence inflammatory cytokine levels.

### 2.5. DTX Effectively Reduces Atherosclerosis Development

Early-stage atherosclerosis in *ApoE*^−/−^ mice typically develops in the aortic root and aortic arch [32,33]. These regions are prone to the development of atherosclerotic plaques due to the complex flow dynamics and susceptibility of lipid accumulation. To investigate whether DTX reduces atherosclerotic lesion formation in these primary sites, we collected the aortic root, including the aortic sinuses, as well as the aortic arch, along with a portion of its three branch arteries: the brachiocephalic, left common carotid, and left subclavian arteries.

**Figure 4:**
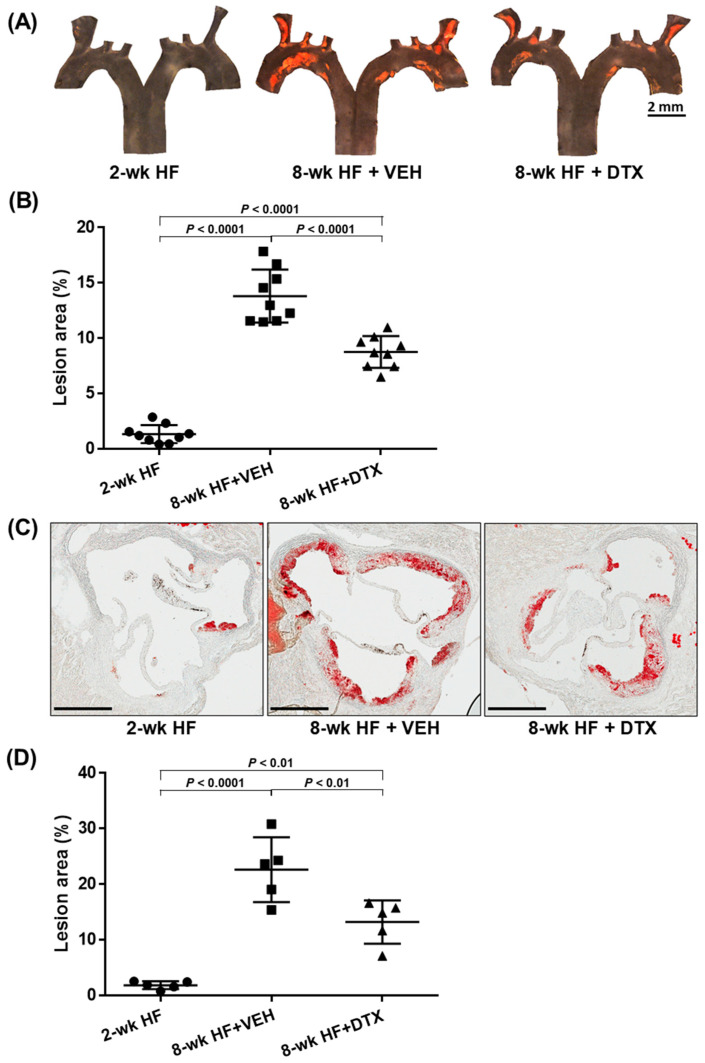
DTX reduces atherosclerosis in the aortic arch (**A**,**B**) and the aortic root (**C**,**D**). (**A**) En face analysis of atherosclerotic lesions in the aortic arch and branch arteries: The aortic arch and its three branches from *ApoE*^−/−^ mice (described in Figure 2) were opened to expose the intimal surfaces, which were stained with Sudan IV to visualize lipid-laden atherosclerotic lesions in orange-red. Representative en face micrographs of the intimal surfaces are shown for the baseline group (2-wk HF), vehicle-treated group (8-wk HF + VEH), and DTX-treated group (8-wk HF + DTX). (**B**) Quantification of lesion area in the aortic arch and branch arteries: The percentage of atherosclerotic legion area was calculated as the ratio of the Sudan IV-stained area to the total intimal surface area. Data are presented as a scatter plot (n = 9) with mean ± standard deviation. Statistical analysis was performed using one-way ANOVA with Tukey’s post hoc test for multiple comparisons. (**C**) Histological analysis of atherosclerotic lesions in the aortic root: Aortic roots from *ApoE*^−/−^ mice (described in Figure 2) were sectioned to obtain cross-sections of the aortic sinus and stained with Oil Red O to visualize lipid-laden atherosclerotic lesions in red. Representative cross-sectional images are shown for the baseline group (2-wk HF), vehicle-treated group (8-wk HF + VEH), and DTX-treated group (8-wk HF + DTX). Scale bar, 400 μm. (**D**) Quantification of lesion area in the aortic root: The total vessel area was determined by tracing the external elastic lamina of the aortic vessel wall. The atherosclerotic lesion area was quantified by measuring the Oil Red O-stained region within the intimal layer of the vessel. The relative lesion area was calculated by subtracting the lesion-free area from the total vessel area and dividing the result by the total vessel area. Data are presented as a scatter plot (n = 5) with mean ± standard deviation. Statistical analysis was performed using one-way ANOVA with Tukey’s post hoc test for multiple comparisons.

The aortic arch was dissected along the coronal plane to expose its intimal surface. Sudan IV staining was then applied to visualize atherosclerotic lesions, which appeared orange-red due to the dye’s binding to the neutral lipids deposited within the lesions. Consistent with previous reports, lesions predominantly developed at the aortic arch and its branch regions, with minimal to no lesions observed in the thoracic descending aorta (Figure 4A). While small lesions were present in the baseline group, the number and size of lesions increased markedly in the vehicle group. However, DTX treatment significantly reduced lesion formation compared to the vehicle group (Figure 4B).

The aortic root was cross-sectioned to obtain sections of the aortic sinuses, which were then stained with Oil Red O to visualize neutral lipids in red. Similar to the findings in the aortic arch, the baseline group exhibited small lesions, while the vehicle group showed a marked increase in both the number and size of lesions (Figure 4C). DTX treatment also significantly reduced lesion formation in these cross-sections compared to the vehicle group (Figure 4D).

These results demonstrate that DTX effectively reduces the accumulation of neutral lipids within the arterial wall and the formation of atherosclerotic lesions. Combined with DTX’s effects on reducing atherogenic lipids and increasing the HDL-C/TC ratio in circulation (Figure 3), these findings strongly support the potential of DTX as a therapeutic agent in mitigating and managing dyslipidemia-induced atherosclerosis.

### 2.6. Limitations

Although sex differences in the pathogenesis of HF diet-induced atherosclerosis in mice are small, these results now need to be replicated in female mice.

While this study focused on evaluating the safety and efficacy of low, non-cytotoxic doses of DTX for short-term treatment, atherosclerosis typically requires long-term management. Therefore, further long-term, longitudinal studies are necessary to assess the sustained efficacy and safety of DTX.

To further investigate the importance of DTX binding to DSC1 in mediating its anti-atherosclerotic effects, we attempted to generate *Dsc1*^−/−^ mice on the C57BL/6 genetic background, commonly used in dyslipidemia and atherosclerosis models such as *ApoE*^−/−^ and *Ldlr*^−/−^ mice. Interestingly, *Dsc1*^−/−^ pups on this background were born at the expected Mendelian ratio but died within the first day of life (data not published). This complication hindered our ability to fully dissect the mechanism of action of DTX.

## 3. Discussion

Despite the existence of preventive and therapeutic measures for ASCVD, such as adopting a healthy lifestyle, smoking cessation, lowering LDL-C with statins, and managing diabetes and hypertension, substantial residual risk remains. ASCVD continues to be the leading cause of morbidity and mortality worldwide, highlighting the need for new therapeutic approaches [34,35]. In the absence of therapies that enhance cholesterol removal from atherosclerotic plaques, we have previously shown that DSC1, a newly identified negative regulator of HDL biogenesis, can be targeted with the FDA-approved chemotherapy drug DTX at non-cytotoxic concentrations to promote HDL biogenesis [2,10]. Given that the RCT pathway, initiated by HDL biogenesis in the arterial wall, is the most well-understood mechanism for cholesterol removal from atherosclerotic plaques, this study investigated whether non-cytotoxic, low-nanomolar concentrations of DTX can reduce atherosclerosis. The results suggest that DTX may be developed as a new class of anti-atherogenic drug that mitigates ASCVD by promoting HDL biogenesis.

Since mammals lack the ability to enzymatically degrade cholesterol, the RCT pathway serves as the primary mechanism for its elimination from the body. In this process, HDL mobilizes cholesterol from extrahepatic tissues, particularly from arterial foam cells, and transports it to the liver, where it is converted into bile acids and excreted through feces [36]. DTX has been shown to promote HDL biogenesis, increase the HDL-C/TC ratio in circulation (Figure 3H), and decrease circulating levels of ALT, a biomarker of hepatic steatosis (Figure 3A). These data suggest that DTX may reduce hepatic cholesterol accumulation by accelerating the RCT pathway. As the complete elimination of cholesterol via this pathway could protect against both hepatic steatosis and atherosclerosis, further investigation into whether DTX modulates hepatobiliary cholesterol excretion would be valuable.

The liver is crucial not only for completing the RCT pathway but also for maintaining whole-body lipid homeostasis, as it acts as the central hub for lipid and lipoprotein metabolism [37,38]. Through the bloodstream, the liver communicates with various organs, including adipose tissue, muscle, the intestine, and the brain, to coordinate lipid synthesis, storage, breakdown, and redistribution in response to changing nutritional and physiological conditions [39,40]. This coordination is reflected in circulating lipid and lipoprotein levels, making the serum lipid profile a recognized indicator of liver function. The improved circulating lipid profile in DTX-treated mice—characterized by reductions in atherogenic lipids (TG, NEFA, TC, and LDL-C) and an elevated HDL-C/TC ratio (Figure 3)—suggests that DTX may enhance hepatic lipid metabolism. The reduction in ALT levels in DTX-treated mice (Figure 3A) further supports a potential hepatoprotective effect of DTX. Investigating whether DTX systemically improves lipid metabolism by modulating the liver’s capacity to regulate lipid synthesis, storage, breakdown, and redistribution would be an interesting avenue for future research.

Our findings indicate that DTX reduces early-stage atherosclerosis by lowering circulating atherogenic lipids (Figure 3) without significantly altering circulating inflammatory cytokine levels (Supplemental Figure S1). These results suggest that inflammation has not yet become a major contributor in early atherosclerosis and that DTX may not directly modulate the immune response at this stage. As the disease progresses, atherogenic lipids and inflammatory cytokines interact dynamically, becoming interdependent drivers of disease progression, In the later stages, inflammation becomes an independent driver of plaque instability and rupture [41,42]. Given their interaction, we cannot exclude the possibility that DTX may influence immune responses during the advanced stages of atherosclerosis. Therefore, further investigation into the effects of DTX in these later stages would be of great interest.

In this study, we utilized the ALZET osmotic pump model 2006 for the controlled delivery of low-nanomolar doses of DTX. However, for the long-term treatment of chronic conditions such as ASCVD, it may be necessary to develop orally administrable formulations of DTX. Although intensive efforts have been made to use the oral route in DTX-based cancer chemotherapy, its low oral bioavailability hinder the delivery of therapeutic doses (1.9–5.1 μM in the bloodstream). Consequently, DTX in chemotherapy is administered intravenously [43,44]. The primary barriers to oral absorption of DTX include poor water solubility, excretion by P-glycoprotein in the gastrointestinal tract and liver, and extensive first-pass metabolism by cytochrome P450 3A4 [45,46]. These pharmaceutical and pharmacological challenges make it difficult to achieve micromolar concentrations via oral delivery. However, these limitations may not necessarily impede the oral delivery of DTX at atheroprotective doses (2.7–4.3 nM in the bloodstream). We are currently exploring various oral formulations of DTX aimed at optimizing bioavailability for these low-nanomolar, atheroprotective doses.

In conclusion, our study has shown that DTX effectively promotes HDL biogenesis, improves the circulating lipid profile, and reduces dyslipidemia-induced atherosclerosis at low-nanomolar concentrations. This potency highlights the potential to repurpose DTX, a drug widely used in chemotherapy, as an HDL-directed atheroprotective therapeutic agent. Statins, the first-line medications for treating dyslipidemia-induced atherosclerosis, primarily work by inhibiting cholesterol biosynthesis [47,48]. In contrast, DTX promotes the removal of excess cellular cholesterol, suggesting that it may act additively or synergistically with statins to reduce cholesterol accumulation in atherosclerotic plaques. Furthermore, while statins can cause mild-to-moderate hepatotoxicity and raise blood glucose levels [48,49], DTX shows hepatoprotective effects (Figure 3A) and lowers glucose levels (Figure 3D). These differences in mechanism of action suggest that DTX could be developed as an add-on or alternative therapy for managing atherosclerosis.

## 4. Materials and Methods

### 4.1. Materials

DMSO, its deuterated form (DMSO-D6), D_2_O, and PEG 300 were purchased from MilliporeSigma (Oakville, ON, Canada). DTX was obtained from Enamine (Cincinnati, OH, USA), and its deuterated form, DTX-D9, from Clearsynth (Toronto, ON, Canada). Implantable mini-osmotic pumps (ALZET model 2006) were acquired from ALZET Osmotic Pumps (Cupertino, CA, USA). The HF diet, consisting of 21% fat and 0.2% cholesterol (Envigo catalog #TD.88137), was purchased from ENVIGO (Madison, WI, USA).

### 4.2. Animal Husbandry

*ApoE*^−/−^ mice (JAX stock #002052) were purchased from The Jackson Laboratory (Bar Harbor, ME, USA) and housed in the Animal Resources Facility at the Research Institute of the McGill University Health Centre (RI-MUHC). The mice were maintained on a 12 h light/12 h dark cycle, fed standard rodent chow (Envigo catalog #2918), and given ad libitum access to water. Room temperature was maintained between 20 and 26 °C, and relative humidity between 40 and 60% via a building automation system. To induce dyslipidemia-driven atherosclerosis, ten-week-old *ApoE*^−/−^ mice were placed on an HF diet (Envigo catalog #TD.88137) for 8 weeks. At the end of the experimental period, the mice were fasted for 4 h prior to euthanasia. The mice were first anesthetized with isoflurane vaporized at a concentration of 4–5% in oxygen, delivered via a chamber until unconsciousness was achieved. This was followed by euthanasia through CO_2_ inhalation. Blood and tissues were then collected for analysis. All procedures were performed in accordance with protocols approved by the Animal Care Committee of the RI-MUHC (Animal use protocol #MUHC-7480), in compliance with the NIH Guide for the Care and Use of Laboratory Animals and ARRIVE guidelines.

### 4.3. Analysis of the Chemical Stability of DTX

To assess the chemical stability of DTX at 37 °C, a solution containing 1 μg/μL of DTX dissolved in a 50% DMSO-D6 and 50% D_2_O mixture was incubated at 37 °C for 6 weeks. Each week, an aliquot of the incubated solution was withdrawn and diluted with DMSO-D6 to achieve a final ratio of 70:30 (DMSO-D6/D_2_O). These samples were analyzed using ^1^H NMR spectroscopy to monitor structural and compositional changes in the DTX compound over the 6-week duration. A Bruker Avance III HD NMR spectrometer, equipped with a 5 mm inverse triple resonance Z-gradient probe and a SampleJet autosampler, was operated at a frequency of 600.17 MHz for ^1^H using Bruker’s Topspin software v3.5pl7 (Billerica, MA, USA). A standard one-dimensional ^1^H NMR spectrum was acquired with water presaturation (50 Hz) during interpulse delays using the Bruker pulse sequence zgpr. A total of 32,000 complex data points were accumulated for 512 scans over a spectral width of 16 ppm using a 1.7 s acquisition delay and a 2 s relaxation delay. The free induction decay signals were apodized by an exponential function, resulting in a 0.3 Hz line broadening, and were then zero-filled to 128,000 data points before Fourier transformation. All spectra were then automatically phased and baseline corrected, before being referenced to the residual two methyl groups of the DMSO signal at 2.50 ppm.

### 4.4. Measurement of DTX Concentration in Mouse Serum

A serum sample (50 μL) was mixed with 5 μL of an internal standard solution (250 ng/mL of DTX-D9) and 0.5 μL of acetic acid. DTX in the mixture was extracted using 1 mL of methyl tertiary-butyl ether. The extract was dried and then reconstituted in a 50 μL solution (methanol/water = 7:3, *v*/*v*) comprising 0.5% sodium acetate at a concentration of 20 μM. The reconstituted sample was sonicated for 10 min, followed by centrifugation at 15,000 rpm for 10 min. The supernatant was then transferred to a glass autosampler vial, from which 10 μL was injected into a liquid chromatography–mass spectrometry (LC-MS) system. The LC-MS analysis was conducted using a triple quadrupole MS system (EVOQ Elite, Bruker, Billerica, MA, USA) coupled with an ultrahigh-performance LC pump (Advance, Bruker). Chromatography was performed on an Agilent Zorbax 300SB-C18 column at 30 °C with a flow rate of 0.4 mL/min, using a gradient solvent system. Mobile phase A (aqueous) consisted of H_2_O with 0.1% acetic acid, while mobile phase B (organic) consisted of methanol with 0.1% acetic acid. The LC method started with 63% B for 1 min, followed by a linear increase to 80% B over 2 min. This gradient was held at 80% B for 2 min before returning to 63% B in 0.1 min, followed by a 3 min conditioning period at 63% B. The total run time per sample was 8 min. The MS operated with the following parameters: ESI source in positive mode, positive spray voltage set to 4000 V, cone temperature at 350 °C, cone gas flow maintained at 20 psi, heated probe temperature set to 400 °C, probe gas flow at 40 psi, and nebulizer gas flow at 60 psi. The MS was used in the multiple reaction monitoring mode. Three transitions were monitored for DTX: 830.3→549.1 (CE 27 eV), 830.3→304.0 (CE 22 eV), and 830.3→248.0 (CE 32 eV), and for DTX-D9: 839.3→549.1 (CE 26 eV), 839.3→312.8 (CE 25 eV), and 839.3→248.9 (CE 34 eV). The concentration of DTX was quantified using an internal calibration curve. A seven-point calibration was employed to generate a standard curve, demonstrating linearity within the dynamic range of 0.4 to 100 ng/mL in mouse serum. The calibration curve was fitted using quadratic regression with a weighting factor of 1/x^2^, showing excellent fit across the calibration range with an R^2^ > 0.99.

### 4.5. Subcutaneous Implantation of Osmotic Pumps

To achieve non-cytotoxic, accurate, and continuous dosing of DTX, we utilized an implantable osmotic pump (ALZET model 2006). The pump was filled according to the manufacturer’s instructions with either DTX at a concentration of 1 μg/μL, dissolved in a solvent mixture comprising 50% DMSO and 50% PEG 300, or with the vehicle solvents alone. The filled pumps were placed in 0.9% saline at 37 °C for 60 h to allow the pumps to reach their steady-state pumping rate before implantation. As part of pre-operative care, a systemic analgesic, carprofen, was administered subcutaneously at a dose of 20 mg/kg 30 min before surgery. The mouse was then anesthetized with isoflurane vaporized at a concentration of 2–3% in oxygen, delivered via a nose cone until surgical unconsciousness was achieved. To prevent corneal desiccation, Optixcare Eye Lube, an ophthalmic ointment, was applied on both eyes. The fur over the surgical area between the scapulae was shaved, and the area was cleaned with 70% isopropyl alcohol followed by 2% chlorhexidine. To prevent dehydration during the procedure, 0.3 mL of 0.9% saline per 10 g of body weight was injected subcutaneously. Once the mouse was properly prepared, a small incision was made between the scapulae for pump insertion, and 1–2 drops of a combined 2% lidocaine and 0.5% bupivacaine solution were applied to the incision site as a local anesthetic. After inserting the pump and closing the incision, the mouse was placed in a heating cabinet to recover until it returned to normal activity. This surgical procedure was conducted in accordance with McGill University’s standard operating procedure #201 for survival rodent surgery (https://www.mcgill.ca/research/research/sop (accessed on 30 March 2023)).

### 4.6. Complete Blood Count

Approximately 150 μL of whole blood was collected into a dipotassium EDTA tube for a complete blood count test. This test included measurements of hemoglobin, hematocrit, mean corpuscular volume, and mean corpuscular hemoglobin concentration, as well as counts of white blood cells, red blood cells, and platelets. The test was conducted in the Diagnostic Laboratory at the Comparative Medicine & Animal Resources Centre, McGill University (Montreal, QC, Canada).

### 4.7. Measurement of Serum Biochemical Parameters Associated with Lipid Metabolism

Blood collected in a serum separator tube was allowed to sit at room temperature for clotting for 30 min, followed by centrifugation at 2000× *g* for 10 min at 4 °C to separate the serum from the cellular components. The serum samples were sent to Charles River Laboratories (Shrewsbury, MA, USA) for the measurement of total cholesterol, free fatty acid, HDL, LDL, TG, ALT, and glucose levels.

### 4.8. Measurement of Cytokine Levels in Plasma

Blood collected in a dipotassium EDTA tube on ice was centrifuged at 2000× *g* for 15 min at 4 °C to separate the plasma from the cellular components. The plasma samples were analyzed for the levels of 25 cytokines, including interleukin (IL)-1β, IL-2, 1L-4, IL-5, IL-6, IL-10, 1L-12p70, IL-13, IL-15, IL-17A, IL-17F, IL-21, IL-22, IL-23, IL-25/IL-17E, IL-27, IL-28B, IL-31, IL-33, CD40 ligand (CD40L), granulocyte-macrophage colony-stimulating factor (GM-CSF), interferon gamma (IFNγ), macrophage inflammatory protein 3 alpha (MIP-3α)/chemokine (C-C motif) ligand 20 (CCL20), and the tumor necrosis factors TNFα and TNFβ. This was performed using a Milliplex Map kit (MilliporeSigma, Cat. No. MT17MAG47K-PX25) according to the manufacturer’s instructions.

### 4.9. Quantification of Atherosclerotic Lesions

Mice were bled and then transcardially perfused with 15 mL of ice-cold phosphate-buffered saline prior to isolating the heart and aorta [50]. A section containing the heart, the aortic arch with its three branches (brachiocephalic, left common carotid, and left subclavian arteries), and the proximal thoracic aorta was collected. The heart was then dissected from the aorta by cutting at the point where the aorta emerges from the left ventricle. Both the heart and the remaining section were fixed in formalin for 48 h at 4 °C. To quantify atherosclerotic lesions in the aortic root, the lower two thirds of the heart were removed. The remaining upper third was immersed in a 30% sucrose solution overnight at 4 °C for cryoprotection. Following this, the tissue was embedded in an optimum cutting temperature compound, snap-frozen, and sectioned at −20 °C to obtain 10 μm-thick cross-sections of the aortic sinus. The sections were then stained with Oil Red O to visualize lipid-laden atherosclerotic lesions in red [51]. The stained sections were mounted in an aqueous medium, and micrographs were captured at 20× magnification using a light microscope. ImageJ software (version 1.54g, downloaded from https://imagej.net/ij/, on 18 October 2023) was used to quantify both the Oil Red O-positive area and the total vessel areas, enabling the calculation of the lesion area percentage [51]. All quantification was performed in a blinded manner. To quantify atherosclerotic lesions in the aortic arch, the arch, along with its three branches and the proximal thoracic aorta, was carefully dissected from the surrounding tissue under a stereomicroscope. The aorta was then incised along the coronal plane to expose its intimal surface. The opened aorta was pinned onto a parafilm bed and stained en face with Sudan IV to visualize lipid-laden atherosclerotic lesions in orange-red [51]. Micrographs of the stained aorta were captured at 7.5× magnification using a stereomicroscope. The lesion area was quantified with ImageJ software and expressed as a percentage of the stained area relative to the total aortic area.

### 4.10. Statistical Analysis

A one-way analysis of variance (ANOVA) followed by Tukey’s post hoc correction was used to compare the means of more than two groups. Multiplicity-adjusted *p*-values were calculated using GraphPad Prism 6 software, with *p* < 0.05 considered statistically significant.

## Figures and Tables

**Table 1:** Complete blood count results of *ApoE*^−/−^ mice.

Component	Reference Range	Non-Treated Group Mean ± SEM (n = 5)	Vehicle-Treated Group Mean ± SEM (n = 5)	DTX-Treated Group Mean ± SEM (n = 5)
WBC count (×10^9^/L)	2–10	7.8 ± 1.48	8.3 ± 1.32	7.7 ± 1.73
RBC count (×10^12^/L)	7.8–10.6	9.8 ± 0.31	9.4 ± 0.27	9.3 ± 0.25
Hemoglobin (g/L)	136–164	145.4 ± 2.50	142.2 ± 3.15	140.2 ± 2.75
Hematocrit (L/L)	0.35–0.52	0.5 ± 0.01	0.5 ± 0.01	0.5 ± 0.01
MCV (fL)	45–55	48.6 ± 1.12	50.4 ± 0.40	50.2 ± 0.58
MCH (pg)	14–17	14.8 ± 0.44	15.2 ± 0.23	15.2 ± 0.18
MCHC (g/L)	300–380	304.6 ± 4.87	301.2 ± 2.20	303.0 ± 2.49
Platelet count (×10^9^/L)	900–1600	1107.8 ± 143.41	1075.8 ± 94.35	1169.2 ± 129.01

WBC, white blood cells; RBC, red blood cells; MCV, mean corpuscular volume; MCH, mean corpuscular hemoglobin; MCHC, mean corpuscular hemoglobin concentration; SEM, standard error of the mean.

## Data Availability

The data supporting the findings of this study are available from the corresponding author upon reasonable request.

## Supplementary Materials

The following supporting information can be downloaded at: https://www.mdpi.com/article/10.3390/ijms26041484/s1.

## Abbreviations and Acronyms

ABCA1, adenosine triphosphate-binding cassette transporter A1; ALT, alanine aminotransferase; ANOVA, analysis of variance; ApoA-I, apolipoprotein A-I; ASCVDs, atherosclerotic cardiovascular diseases; CCL20, chemokine (C-C motif) ligand 20; CD40L, CD40 ligand; DSC1, desmocollin 1; D_2_O, deuterium oxide; DMSO, dimethyl sulfoxide; DTX, docetaxel; FDA, Food and Drug Administration; GM-CSF, granulocyte-macrophage colony-stimulating factor; HDL, high-density lipoprotein; HDL-C, HDL cholesterol; HF, high fat; IFN, interferon; IL, interleukin; LDL, low-density lipoprotein; LDL-C, LDL cholesterol; LC-MS, liquid chromatography–mass spectrometry; MIP-3α, macrophage inflammatory protein 3 alpha; NEFA, non-esterified fatty acid; PEG, polyethylene glycol; ^1^H NMR, proton nuclear magnetic resonance; RCT, reverse cholesterol transport; SMCs, smooth muscle cells; TC, total cholesterol; TGs, triglycerides; TNF, tumor necrosis factor; VEH, vehicle; WK, week.
