# Supplementary material for: Low-Dose Docetaxel Is Effective in Reducing Atherogenic Lipids and Atherosclerosis
Source: Int J Mol Sci. 2025 Feb 11;26(4):1484. doi: 10.3390/ijms26041484 (PMC11855627; doi:10.3390/ijms26041484)
Supplement: Supplementary file 1 [file ijms-26-01484-s001.zip › ijms-3447185-supplementary.pdf]

Supplemental Figures

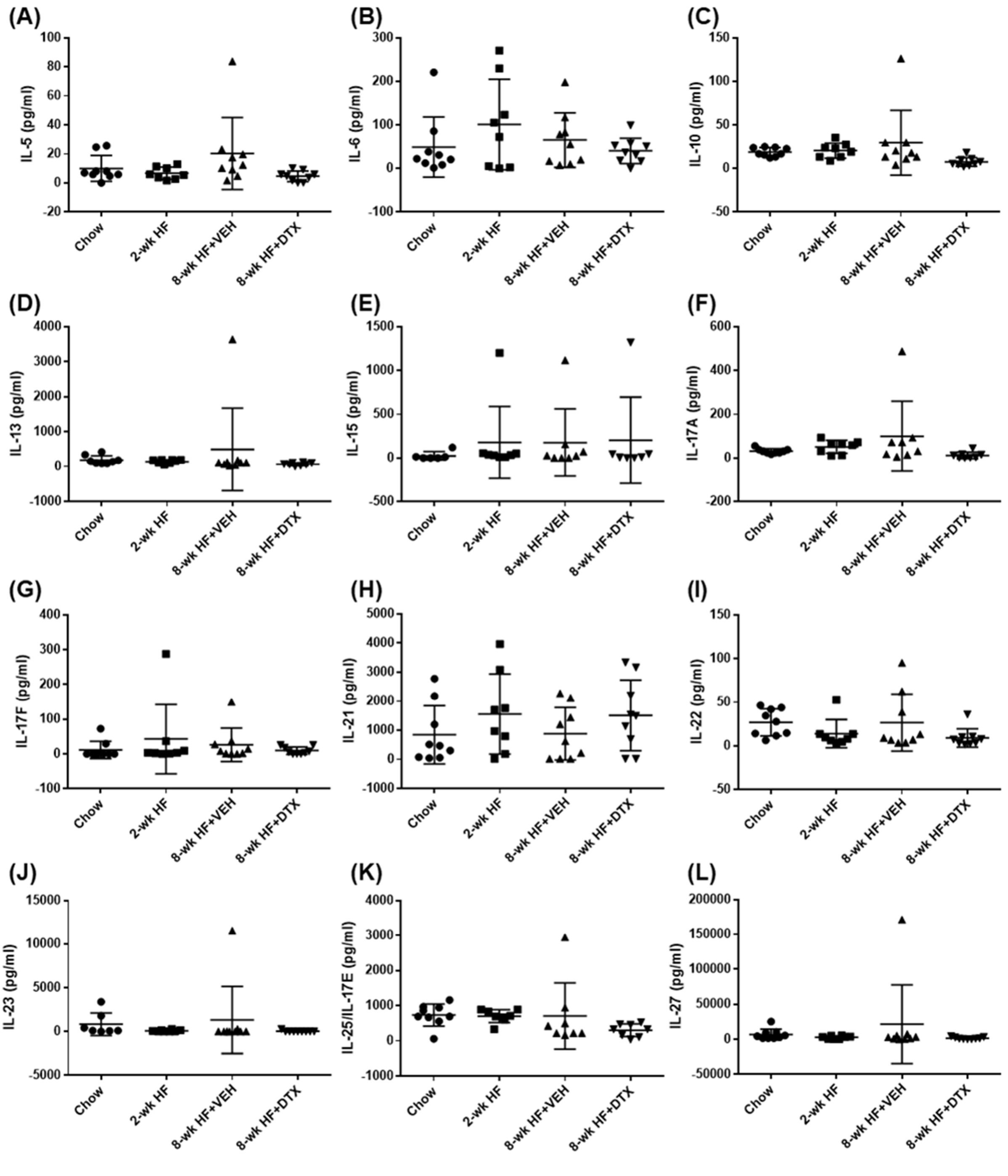

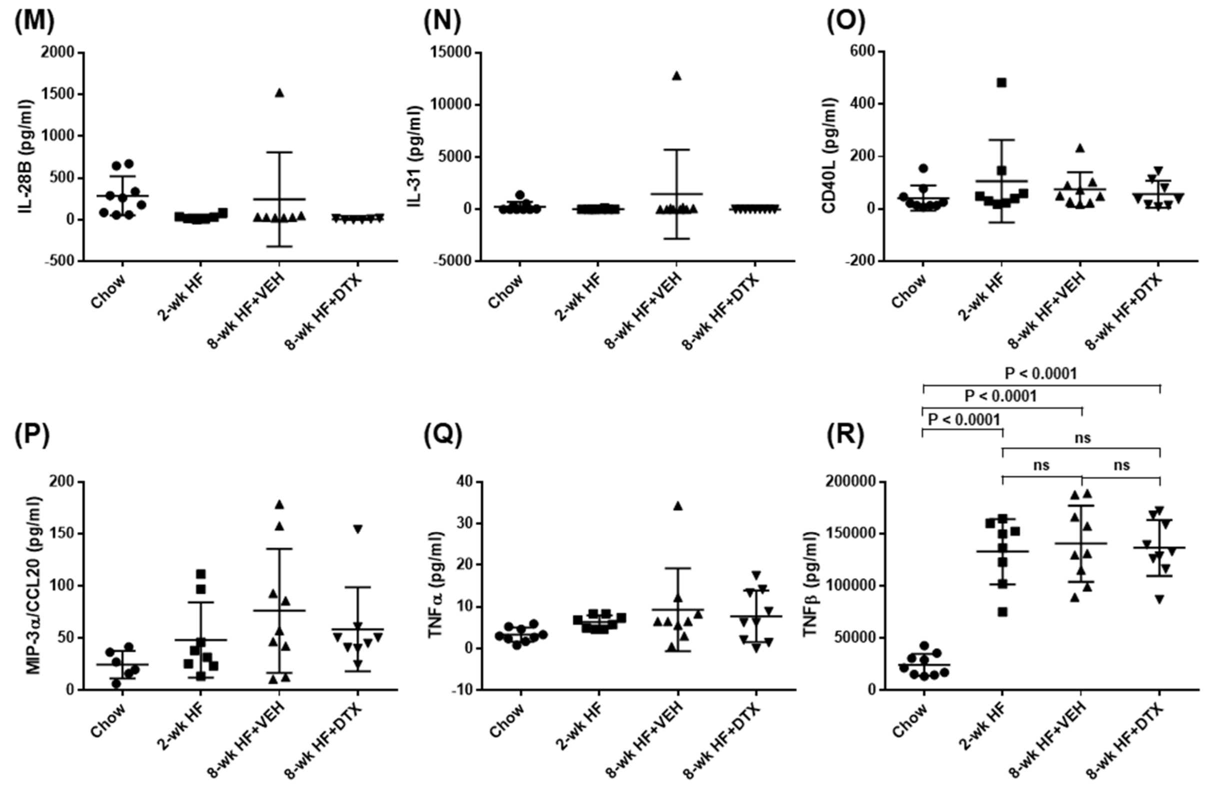

Figure S1. Plasma levels of cytokines in *ApoE*$^{-/-}$ mice. Ten-week (wk)-old *ApoE*$^{-/-}$ male mice were fed a high-fat (HF) diet and treated according to the timeline outlined in Figure 2 of the main manuscript. Blood samples were collected from chow-fed mice (immediately before starting the HF diet), baseline group mice (2-wk HF), vehicle (VEH)-treated mice (8-wk HF + VEH) and docetaxel (DTX)-treated mice (8-wk HF + DTX). Plasma samples, separated from the blood, were analyzed for circulating levels of the indicated cytokines: IL-5 (A), IL-6 (B), IL-10 (C), IL-13 (D), IL-15 (E), IL-17A (F), IL-17F (G), IL-21 (H), IL-22 (I), IL-23 (J), IL-25/IL-17E (K), IL-27 (L), IL-28B (M), IL-31 (N), CD40L (O), MIP-3a/CCL20 (P), TNFa (Q) and TNF b (R). Data are presented as scatter plots (n = 6 - 9) with mean ± standard deviation. Statistical analysis was performed using One-way ANOVA with Tukey's post hoc test to adjust for multiple comparisons, with $P < 0.05$ considered statistically significant. Values marked as "ns" indicate not significant difference. No statistically significant differences were observed in Figures 1A-1Q.
